# Inverse Design of Highly Deformable Mechanical Metamaterial Based on Partitional Semi‐Random Optimization

**DOI:** 10.1002/advs.202415935

**Published:** 2025-05-20

**Authors:** Xueqing Cao, Zeang Zhao, Panding Wang, Shengyu Duan, Hongshuai Lei

**Affiliations:** ^1^ Beijing Key Laboratory of Lightweight Multi‐functional Composite Materials and Structures Beijing Institute of Technology Beijing 100081 P. R. China

**Keywords:** flexible structure, inverse design, mechanical metamaterial, topology optimization

## Abstract

The flexible mechanical metamaterial leverages the reversible large deformation of its lattice unit cells to transfer force, motion, and energy. This characteristic imparts significant mechanical advantages for both morphological and functional regulation. However, most existing topology optimization methods primarily focus on initial stiffness and degrees of freedom under small deformations, which are incapable of the customization of large deformations in mechanical metamaterials. In this research, the concept of partitional semi‐random optimization is proposed, which respectively records the structural evolution history in each subregion and evaluates the value. The new concept involves a statistical decision‐making procedure, which avoids the repetitive iterations in traditional completely random heuristic optimizations. In addition, a two‐step optimization scheme for local stiffness regulation is developed to expand the design space of one single material. This method facilitates the personalized customization of both the multi‐objective deformation pattern and the deformation path of highly deformable mechanical metamaterial made from homogeneous rubbers. The efficiency of the design process is verified by experiments with 3D‐printed metamaterial samples. This research offers a novel solution to the large deformation design of shape‐morphing structures and soft robotics.

## Graphical abstract

This research presents a novel optimization method for the inverse design of flexible mechanical metamaterials, enhancing traditional strategies by evaluating deformation in subregions. Combining simulated annealing with a genetic algorithm, it improves design efficiency and enables customizable deformation paths. Experimental validation confirms its effectiveness, with potential applications in biomedical devices and adaptive structures.

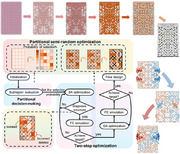

## 1 Introduction

The flexible mechanical metamaterial utilizes the reversible large deformation of the unit cell to transfer force, motion, and energy,^[^ ^1^ ^]^ and has the mechanical properties of both structure and mechanism.^[^ ^2^ ^]^ One of the most important advantages of the flexible lattice structure is that it can flexibly adjust its mechanical properties and deformation modes by changing the topological parameters, so as to adapt different application scenarios in the optimal structural form.^[^ ^3^ ^]^ The flexible mechanical metamaterial uses the lattice unit cells to replace local beam, plate, and shell components of traditional compliant mechanism,^[^ ^4^ ^]^ which further improves the mechanical properties of the flexible structure, including: wider designability,^[^ ^5^ ^]^ higher degrees of freedom,^[^ ^6^ ^]^ better reliability.^[^ ^7^ ^]^ The design of macro‐topology and micro‐cell configuration is an indispensable step to realize the practical application of flexible mechanical metamaterial.^[^ ^8^ ^]^ Recent progress in this field includes the forward design with theoretical analysis and the inverse design with numerical optimization.

The forward design of flexible mechanical metamaterial typically involves arranging unit cells in 3D space according to specific rules, to achieve an overall deformation pattern that meets design objectives. For instance, surface morphology‐programmable flexible metamaterial has been proposed by regulating the spatial arrangement of anisotropic flexible units based on the superposition of unit spin vectors and connectivity constraints.^[^ ^9^ ^]^ Additionally, the local stiffness of flexible metamaterial has been adjusted by varying the diameter of micro‐beams and the size of Voronoi units, which has enabled the automatic design of compliant grippers and gimbals.^[^ ^10^ ^]^ Graph‐based methods have been used to quickly analyze the degrees of freedom for planar flexible structures with quadrilateral units and to automatically determine the unit arrangement based on target motion paths.^[^ ^11^ ^]^ The Freedom and Constraint Topologies (FACT) method, originally used for compliant mechanisms, has been extended to flexible mechanical metamaterials consisting of member and plate units.^[^ ^7^ ^]^ Overall, existing forward design methods for flexible mechanical metamaterials largely continue the degree‐of‐freedom analysis and synthesis approaches used for traditional multi‐body systems. However, it is important to note that the basic component of a multi‐body system is a kinematic pair formed by rigid body contact, with its degree of freedom typically independent of motion stroke. In contrast, for flexible mechanical metamaterials, overall flexibility and degrees of freedom can change significantly under large deformations, indicating that the aforementioned methods may not align with the principles of degree‐of‐freedom analysis and design theory.

Inverse design methods such as structural topology optimization which are widely used in traditional load‐bearing structures,^[^ ^12^ ^]^ have been gradually extended to the design of flexible mechanical metamaterials in recent years. Topology optimization based on triangular grids has enabled the design of execution efficiency of moderate‐deformation flexible structures.^[^ ^13^ ^]^ The stochastic topology optimization through simulated annealing is developed to enhance critical buckling strain and load capacities of flexible beam structures.^[^ ^14^ ^]^ The SIMP (solid isotropic microstructure with penalization) method has been applied to optimize the micro‐orientation of lattice cells, particularly in the context of underactuated soft underwater vehicles.^[^ ^15^ ^]^Additionally, a design strategy that divides the domain into periodic sub‐domains aims to optimize cell configurations and arrangements, has been utilized to design the overall flexibility.^[^ ^16^ ^]^ Furthermore, optimization methods based on the homogenization of cell properties and heterogeneous materials have been developed for flexible metamaterials.^[^ ^17^ ^]^ Very recent advances include the sequential subdomain optimization method which integrates recurrent neural networks with optimization techniques,^[^ ^18^ ^]^ and the “Deep‐DRAM” method which integrates deep learning models with deep generative models.^[^ ^19^ ^]^ Despite these advancements, existing inverse design methods primarily focus on stiffness characterization under small deformations and global stress–strain relationships. They exhibit critical limitations in addressing large deformation customization for flexible mechanical metamaterials, particularly manifested in parasitic motions and irrational deformation patterns during large‐strain operations. Furthermore, gradient‐based optimization algorithms and heuristic approaches face prohibitively high computational costs when solving large‐deformation optimization problems due to the complex nonlinear coupling between material hyperelasticity and structural snap‐through behaviors. These constraints highlight the need for more robust data‐driven frameworks capable of bridging multiscale.

**Figure 1:**
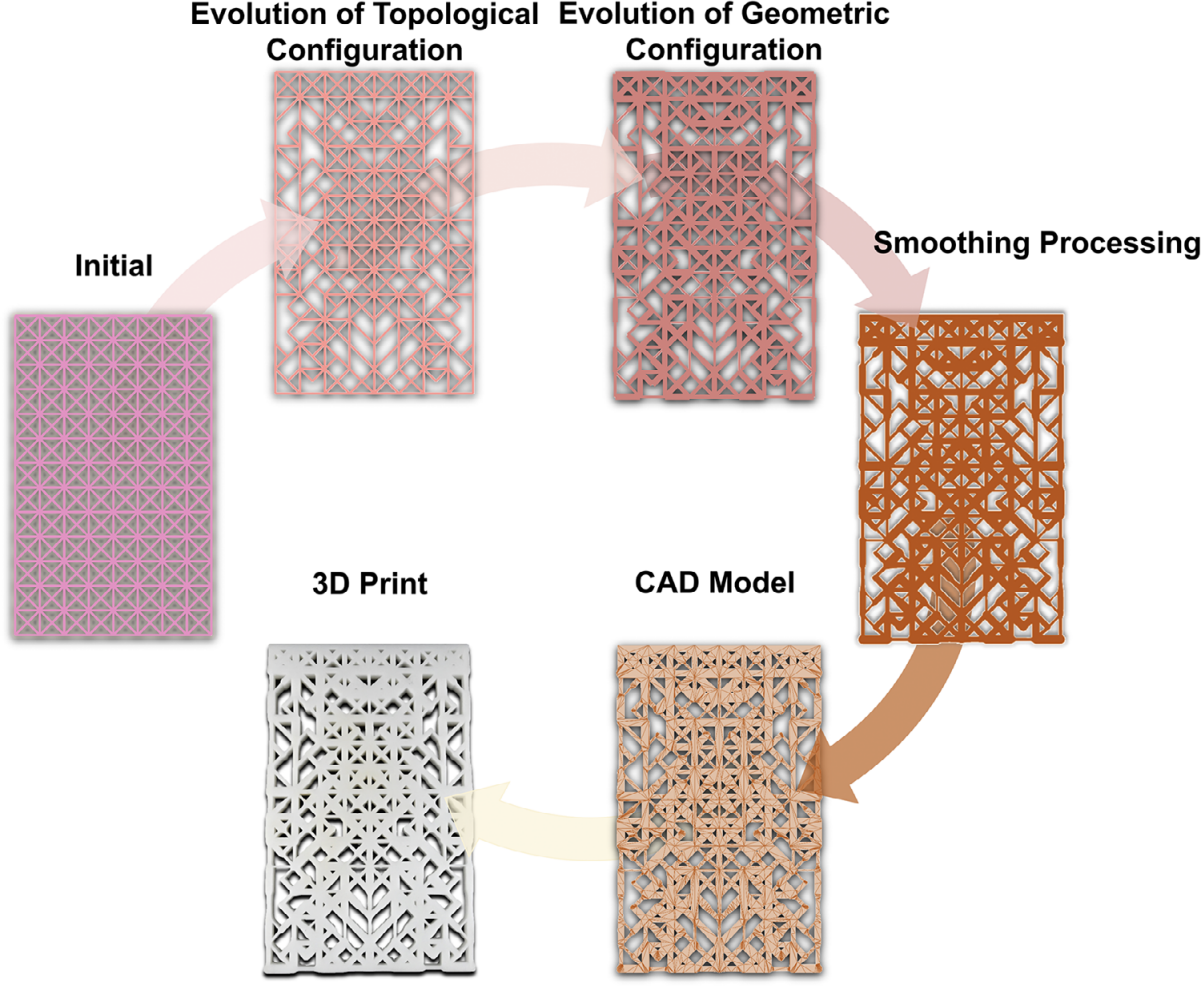
Schematic of a complete inverse design process. The inverse design of highly deformable mechanical metamaterial encompasses the evolution of the structure's configuration by optimization, the transformation from beam model to solid model through geometric smoothing, the creation of a printable CAD model, and the validation through 3D printing and experiment.

In this research, we propose a novel concept termed partitional semi‐random optimization for the inverse design of flexible and highly deformable mechanical metamaterials. This approach systematically records the structural evolution across distinct subregions of the metamaterial, facilitating a comprehensive evaluation. The results of this evaluation guide the selection of subregions for the subsequent optimization process, where they serve as a basis for defining subregion selection probabilities in the heuristic algorithm. By integrating a simulated annealing algorithm with a genetic algorithm within a two‐step optimization scheme focused on local stiffness adjustment, our method also significantly enhances the efficiency of the optimization process. This integration reduces the need for redundant iterations typical of traditional heuristic optimizations, thereby accelerating the attainment of target large deformation. Compared to traditional deep learning methods, our approach eliminates the dependence on large‐scale labeled data through physics‐based learning, offering advantages in computational efficiency and resource utilization. It is particularly effective in achieving rapid optimization iterations in the initial stages. Furthermore, this framework expands the design space available for single‐material, enabling personalized customization of both the multi‐objective deformation pattern and the deformation path of flexible mechanical metamaterials fabricated from homogeneous rubbers. The modifications in the structure within the design‐manufacturing process are illustrated in **Figure** **1**. Our approach allows for reversible large deformations and the realization of continuous curved shapes, providing greater design flexibility to meet the specific requirements of various applications in soft robotics and adaptive structures. To validate the effectiveness and efficiency of our design process, we conduct experiments using 3D‐printed metamaterial samples. The results demonstrate the potential of our method to facilitate large deformation designs in shape‐morphing structures, offering a solution to the challenges faced in this evolving field of metamaterials and their diverse applications.

## 2 Results and Discussion

### 2.1 Partitional Semi‐Random Optimization Algorithm

**Figure 2:**
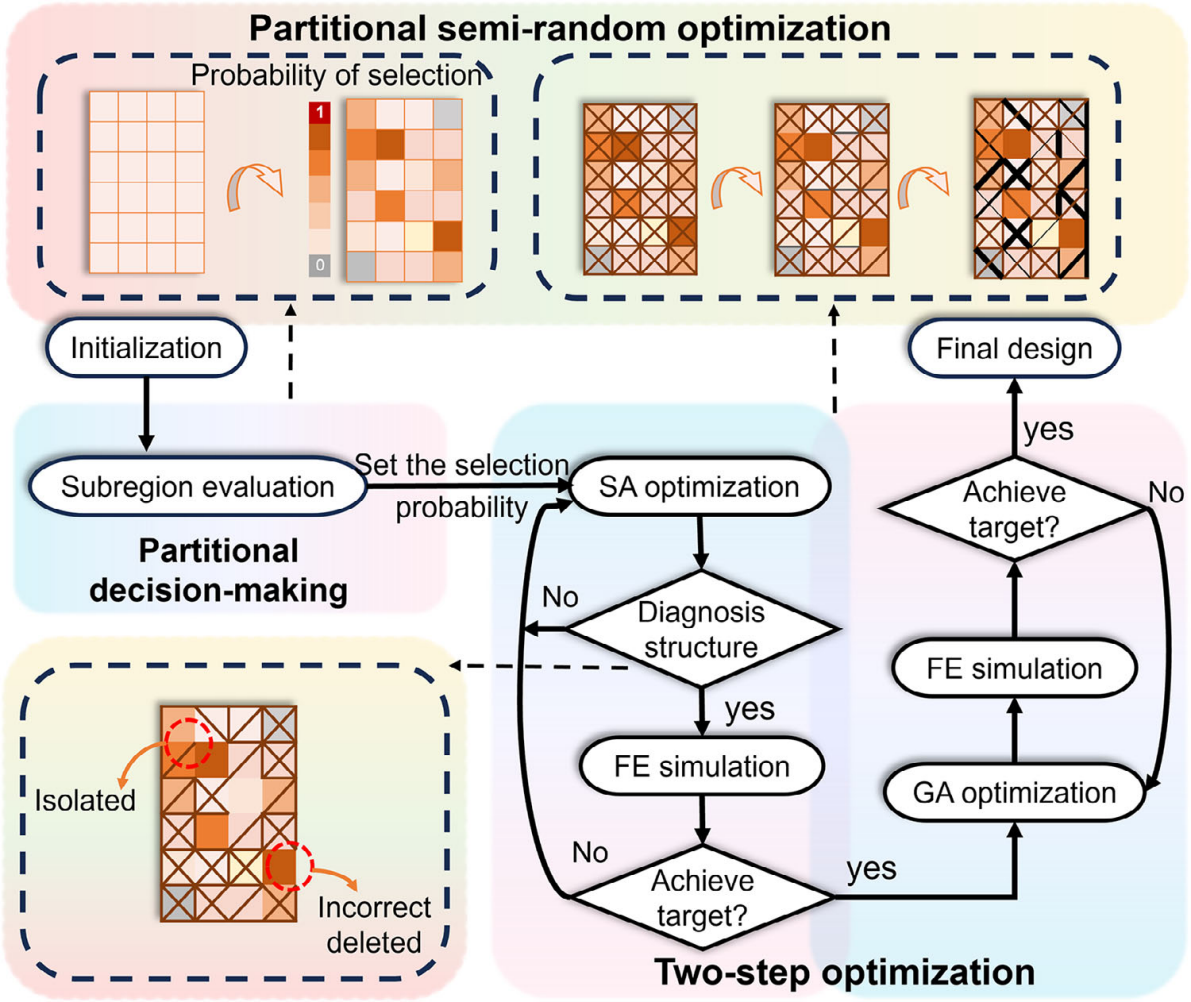
Schematic of the partitional Semi‐random optimization approach. The approach comprises “partitional decision‐making” and “two‐step optimizations.” The optimization begins by defining the initial configuration and deformation target. Next, the structure's effectiveness in different subregions is assessed, and the probability for subsequent statistical decision‐making is determined. In each optimization iteration, the simulated annealing (SA) for topological evolution and the genetic algorithm (GA) for geometric modifications are implemented, until the deformation target is achieved. The dashed box represents the corresponding adjustment and evolution of the structure from the initial configuration to the final design in the relevant steps.

The proposed inverse design process, illustrated in **Figure** **2**, encompasses two key steps: partitional decision‐making and two‐step optimization. First, we establish the initial configuration, define the deformation target, and determine the processing sequence. Subsequently, we assess the effectiveness of the structure in different subregions based on the deformation target and determine the probability of each subregion being selected in the next optimization iteration. Based on the probability distribution established in the previous step, the structure undergoes iterative evolution through a two‐step optimization process. This process includes iterative modifications of the topological configuration using a simulated annealing (SA) algorithm and the geometric configuration using a genetic algorithm (GA), continuing until the design objectives are met. The finite element method is employed to evaluate the consistency with the specified target at each iteration. During the component removal process, the structure's rationality may be altered, thus a model validation is diagnosed to ensure the structural integrity is preserved (Section S1.1, Supporting Information).

The target deformation of the structure can be designed according to different usage requirements. We use a grid‐like structure as the initial model (Figure 1S) and define the target displacement for the reference points where deformation is expected. These subregions are determined based on the initial configuration, aiming for a roughly equal distribution of members within each subregion. The overall size of the structure is typically 20–50 times larger than the individual subregions. The initial structure is divided into several subregions, each containing an equal number of members, and each time members within a subregion are selected for randomly deleted. Importantly, these subregions are connected, ensuring structural continuity, and each member belongs to only one subregion, avoiding redundancy. The efficiency of structural modification in each subregion and during each iteration is assessed by calculating the difference between the temporary deformation and the target deformation. The difference of the selected subregion structure after a single modification is described by the Equation (1):

(1) $$d=\sum_{k=1}^{K}\left(\frac{{\left(H_{k}-H_{k}^{*}\right)}^{2}}{\bar{H}}\right),k=1,\cdots ,K$$

where *H_k_* and *H_k_* *are the temporary deformation and target deformation at selected reference points 𝑘, respectively, 𝐾 is the total number of reference points, $\bar{H}$ is the mean target displacement.

By judging and integrating the difference between the temporary deformation and target deformation of the randomly deleted members in each subregion *D*:

(2) $$D=\frac{\sum_{i=1}^{m}d_{i}}{n},i=1,\text{…},m$$

where*d_i_* is the difference between the temporary deformation and the target deformation at the *i*‐th deletion; *m*is the number of random deletions in this subregion, we can get the selection probability*P_j_*:

(3) $$P_{j}=\frac{D_{j}}{\sum_{j=1}^{n}D_{j}}.j=1,\text{…},n$$

where*D_j_* is the overall difference of the *j*‐th subregion,*n*is the number of all subregions.

By comparing and integrating the selection probabilities of each subregion, we calculate the frequency of selected members and the number of deleted members within each subregion of the overall structure. This information is then used to prioritize optimization based on the high‐probability subdomains identified; by comparing and processing the different selection probabilities of each subregion, we can calculate the frequency of selected members and the number of deleted members, enabling the structure to reach the ideal target state within fewer iterations. This partitional approach is necessary because it allows us to focus computational effort on the most promising areas of the structure. By analyzing the impact of modifications within each subregion, we can identify those that contribute most significantly to achieving the target deformation.

**Figure 3:**
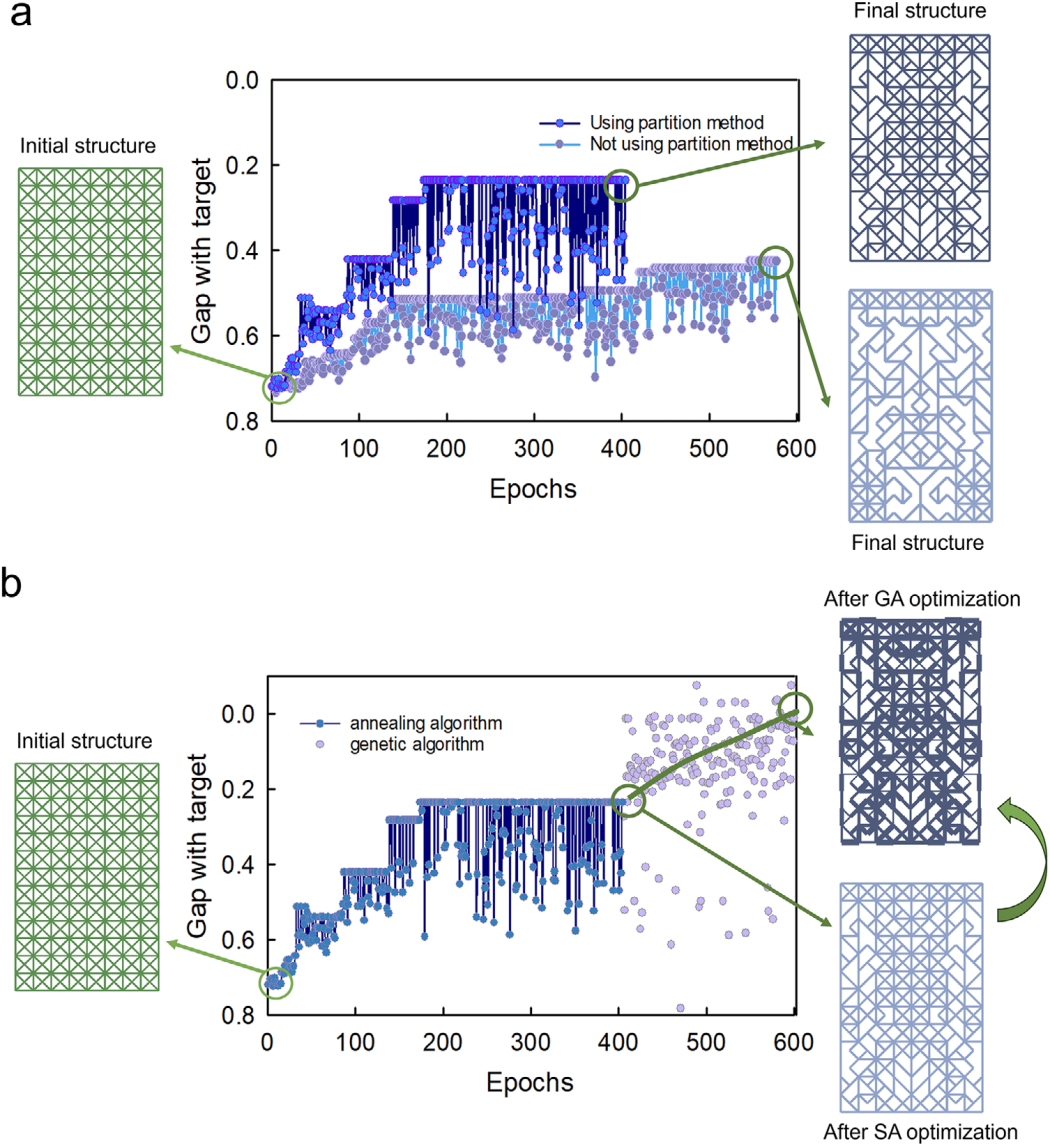
The convergence process for each optimization method a) Comparison of the optimizations with and without using the partitional decision‐making approach. The green image shows the initial structures, while the dark blue and light blue image shows the optimized structures of different methods. b) Comparison of the optimizations with and without using the two‐step optimization. The dark blue and light blue image shows the optimized structures of different methods.

Under the probability distribution established through the Partitional decision‐making process, optimization is carried out by modifying the structure. Specifically, structural members are removed based on their assigned probabilities, leading to a change in the topological configuration. The gap between the modified structure's response and the target value is then calculated. The annealing algorithm is employed to decide whether the modified result should be retained. This iterative process continues until the gap stabilizes and no longer changes. The benefits of the statistical decision‐making procedure are shown in **Figure** **3**a, where we achieve our desired structural performance more efficiently compared to traditional optimization processes.

Subsequently, an additional step is introduced to further reduce the gap: the genetic algorithm (GA) is applied to optimize the geometric configuration. In this phase, the section width of the members within these structures is randomly adjusted. These modifications are then screened and mutated based on performance outcomes, allowing for progressive refinement. This two‐step optimization approach—consisting of topological changes via simulated annealing and geometric adjustments via genetic algorithms—enables continuous improvement of the design. The inclusion of the GA step is particularly beneficial when the optimization process reaches a bottleneck. By allowing for geometric adjustments, the design space is expanded, enabling the structure to more effectively achieve the target performance. As illustrated in Figure 3b, by adjusting both the topological and geometric configurations, the structure can be fine‐tuned to more closely match the target value, ultimately enhancing its overall performance.

### 2.2 Inverse Design of Rectangular Structure with Different Customized Deformation Patterns

In this section, we design a 2D grid structure consisting of 8 × 13 square unit cells (see Figure S1, Supporting Information). The lattice initially comprises 645 adjustable members, with each unit cell having a side length *L* = 8.0 mm and a central point positioned at its center. Consequently, the lengths of the horizontal and vertical members are *l* = *L*, while the diagonal members measure *l* = (√2/2) × *L*. The design includes a mechanism for customized deformation to assess the reliability of the proposed design methodology.

**Figure 4:**
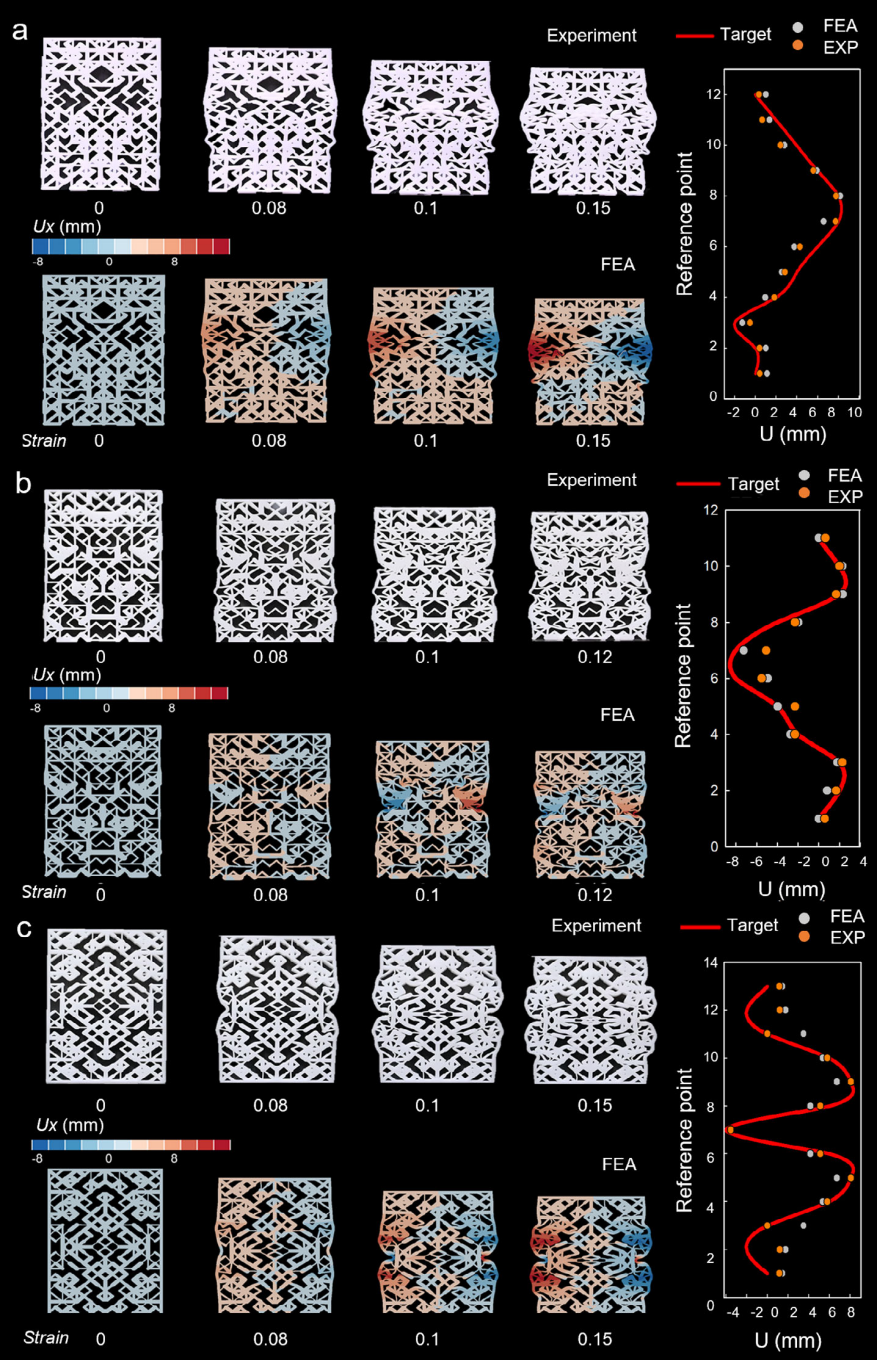
The inverse design of different customized deformation patterns. On the left‐hand side, the deformation process of structures under compression is shown, with the experimental results (depicted by the white prototype) alongside the FEA (Finite Element Analysis) simulation results (represented by the red‐blue deformation contour). On the right‐hand side, a comparison is made between the target deformation pattern, the simulation results, and the experimental results, allowing for a clear evaluation of the accuracy and effectiveness of the inverse design methodology. a–c) represent three different deformation patterns.

Herein, we propose three different shapes of deformation patterns as examples. The corresponding target displacement is set to the reference points on the side of the rectangular structure. After the optimization, as shown in **Figure** **4**, these model structures are capable of producing the desired deformations.

In the first structure, we expect the side to be deformed into a convex shape after being compressed. Considering the boundary conditions of the structure, we set the displacement values that we hope to reach (Figure S1, Supporting Information) for the 12 reference points in the middle edge. To ensure consistent deformation on both sides, we apply symmetric conditions to the structure. This symmetry allows us to optimize only half of the initial model, thereby reducing the number of designed members from 645 to 329. Following these, we obtained the optimal results, which agree well with the target. The inversed‐designed model is 3D printed and experimentally verified on the universal test machine. As shown in Figure 4a, with increasing compression, the lateral deformation becomes increasingly pronounced. When the strain reaches 0.15, the lateral deformation attains a stable configuration. Given that the structure is symmetrical, the deformation observed on both sides remains highly consistent. The deformation results from both the finite element simulations and experimental tests of the final structure were highly favorable, showing significant congruence with the predetermined target shape. Furthermore, the observed convex deformation isn't simply material‐driven; it's dictated by the optimized lattice structure. The design concentrates stress centrally, inducing controlled bending outward. The concave curve is achieved by strategically weakening the central region of the lattice, promoting inward bending. Reinforced convex ends resist inward deformation, creating the desired profile. This demonstrates controlled manipulation of bending via stiffness distribution.

In addition, we have designed another more intricate concave curve shape, as shown in Figure 4b. To differentiate this concave curve from the characteristic behavior of materials with a negative Poisson's ratio, we introduce convex deformation at both ends. This target behavior is distinct from the simple inward contraction typically observed in negative Poisson's ratio materials and demonstrates enhanced controllability in structural design. The final model is a centrosymmetric configuration. We also design one‐quarter of the structure to exhibit a wavy deformation, which is then mirrored to form the complete structure for validation. As depicted in Figure 4c, the side deformation of the structure manifests as a centrosymmetric shape. The integration of simpler curves into a more complex curvature enriches the design, highlighting the versatility of the flexible lattice structure. This approach underscores the potential for designing and assembling various structures to achieve more complex deformation patterns. The integration of simpler curves into a more complex curvature enriches the design, highlighting the versatility of the flexible lattice structure. This approach underscores the potential for designing and assembling various structures to achieve more complex deformation patterns. The wavy deformation further showcases the design's ability to create complex stress distributions by modulating lattice stiffness, resulting in alternating convex and concave regions. This precise control over deformation is a key advantage.

### 2.3 Inverse Design of Deformation Path for Morphing Wings

**Figure 5:**
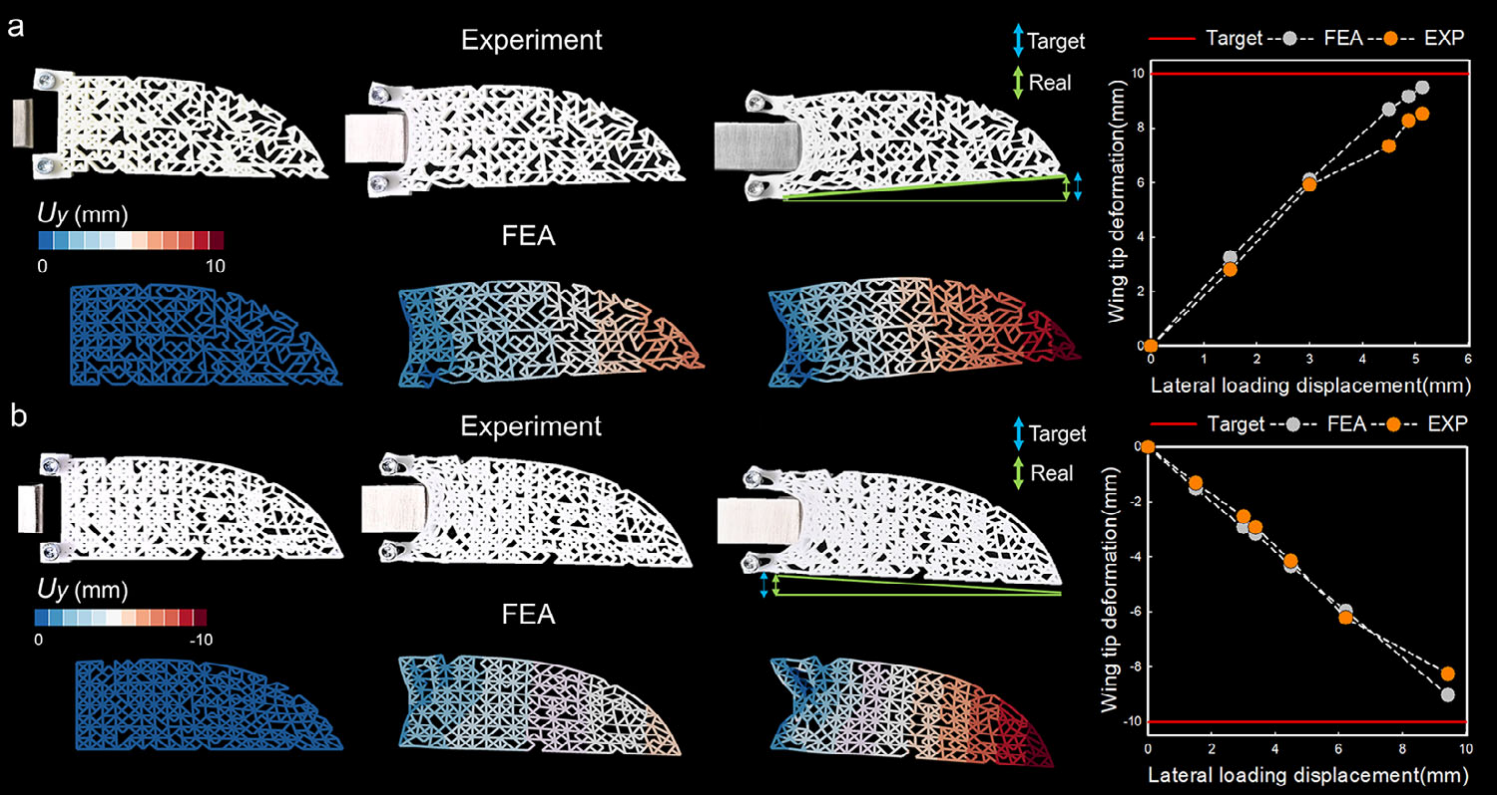
The simulation and experiment verification of the inverse design of the deformation path. a) The wing tip moves upward by 10 mm after the wing root is driven; b) The wing tip moves down 10 mm after the wing root is driven. The white prototype represents the experimental results, while the displacement contours correspond to the finite element simulation results. On the right‐hand side, the relationship between the lateral load displacement and the vertical deformation of the wing tip and a comparison of the differences between simulated and experimental data.

In this section, we mainly propose the application of inverse design of the deformation path. Different from the previous section for the overall deformation pattern of the structure, we mainly focus on the deformation of specific parts of the structure after being driven along the designed path. To illustrate the versatility and broad applicability of this approach to complex geometries, we introduce a wing‐shaped structure, which presents greater complexity and irregularity than a conventional rectangular form. The uniformly distributed square unit cells discussed earlier are not ideal for such intricate shapes. The design process initiates with the definition of the peripheral contour of the structure. Subsequently, the interior of the structure is meshed into quadrilateral elements. Given the contour dimensions of ≈165 × 70 units, the mesh size is constrained to a range between 2 and 9 mm. This selection ensures a judicious balance between computational accuracy and efficiency. A specialized program is then utilized to generate square unit cells that emulate a rectangular arrangement. However, due to the inherent complexity of the structure, the lengths and angles of the members within each cell are adjusted to accommodate varying geometries. This methodological framework enables the creation of lattices for a wide array of complex structures, thereby significantly expanding the potential design space and enhancing the versatility of structural applications. As illustrated in **Figure** **5**, we establish two distinct targets for the morphing wing design: the ability for the wing tip to deflect 10 mm downward or 10 mm upward under lateral loading conditions. In this model, we do not employ the method of varying member section width. This choice is based on two primary considerations: first, since the design primarily focuses on the realization of the deformation path, removing members emerges as a direct and effective method for achieving the specified objectives; second, altering member section width could adversely affect the structural integrity of the connections between irregularly shaped members. Figure 5a depicts the configuration for the upward tip displacement target, while Figure 5b illustrates the configuration for the downward tip displacement target. Both configurations are rigorously validated through comprehensive finite element simulations and experimental testing, ensuring a robust assessment of their performance. Under lateral loadings, the tip displacement effects are notably pronounced, demonstrating a high degree of responsiveness and stability. A more detailed schematic of the lateral driven displacements and wing tip deformations, including a comparative visualization, is available in Figure S4 (Supporting Information). These configurations not only meet the specified targets efficiently but also exhibit enhanced structural behavior, indicating their effectiveness in real‐world applications.

In practical applications, the state of the wing can be effectively controlled in response to the applied loads, enabling it to adapt to various operational conditions. This control method is both efficient and straightforward, relying on the regulation of the integrated structure rather than employing complex mechanical components. As a result, this approach aligns well with the lightweight requirements of modern aviation, facilitating enhanced performance without compromising structural integrity.

### 2.4 Inverse Design of Surface Deformation Patterns for Engine Nozzle

The engine nozzle is a critical component in both jet engines and rockets, serving the essential function of directing exhaust gases to produce thrust. Its primary role is to accelerate these gases, converting thermal energy into kinetic energy, thereby propelling the vehicle forward. The shape and design of the nozzle can significantly influence thrust efficiency, speed, and fuel consumption. In this context, we explore the potential of the flexible mechanical metamaterial with controllable deformation applied to the engine nozzle. The proposed design involves a variable‐shaped nozzle that adjusts its geometry according to the engine's operational requirements. This dynamic adjustment is particularly beneficial during different stages of engine operation, allowing for improved performance and efficiency. Specifically, the nozzle's internal shape can be altered in response to actuation loads, leading to modifications in airflow and thrust characteristics.

**Figure 6:**
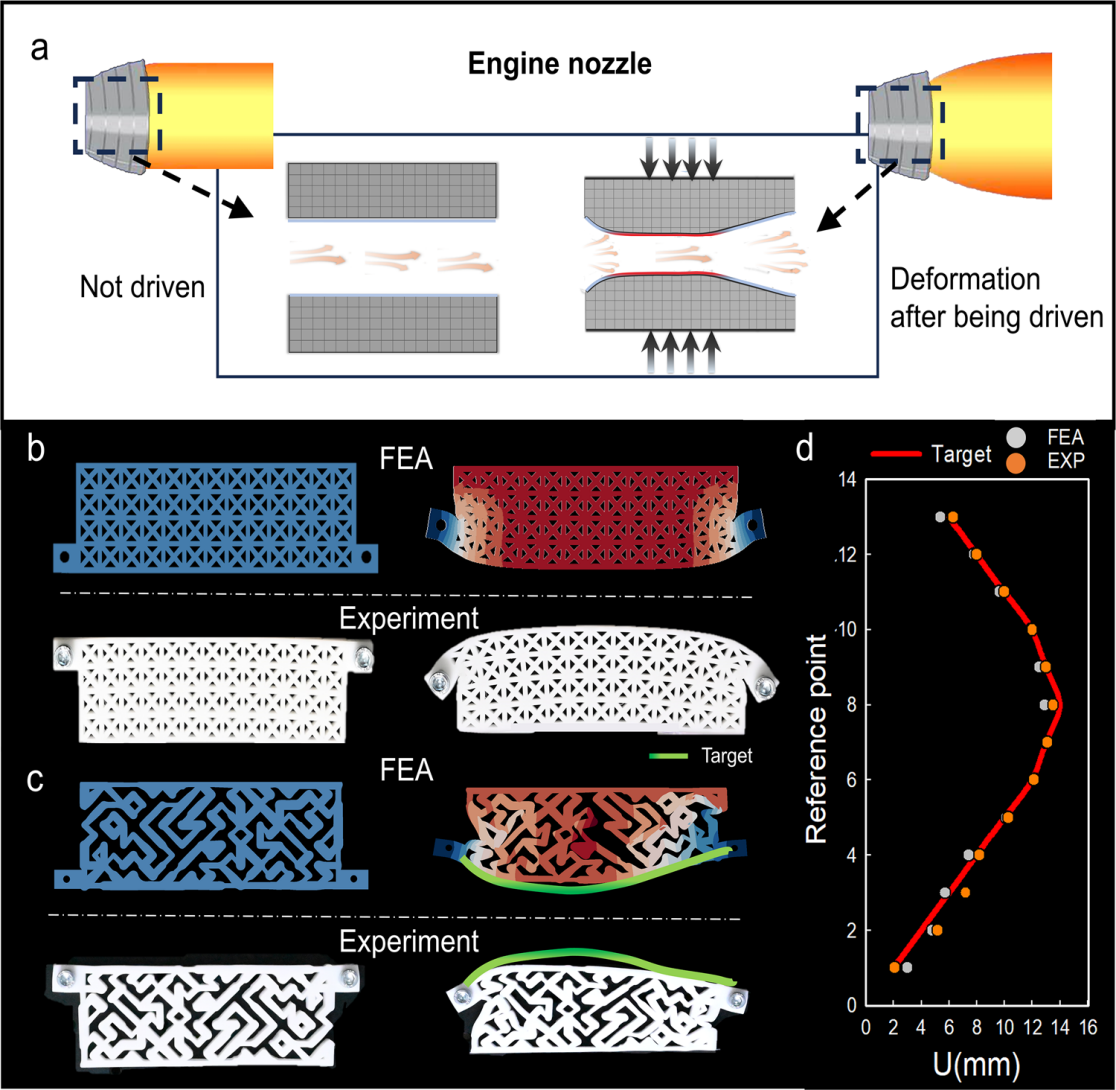
Inverse design of deformation patterns for the engine nozzle. a) The potential application of highly deformable mechanical metamaterial: the shape of the engine nozzle is adjusted by applying a load to adjust the flow properties under different flying conditions. b) Simulation (depicted by the blue‐red deformation contour) and experimental (depicted by the white prototype) results of a homogeneous lattice metamaterial c) Simulation and experimental results of the optimized flexible metamaterial. d) Comparison of the target deformation pattern, the simulation results, and the experimental results.

As illustrated in **Figure** **6**a, the nozzle is designed to operate in two distinct states. In the absence of external loading, the nozzle retains a standardized horizontal configuration, facilitating unobstructed gas flow through the engine. However, when an actuation load is applied, the internal geometry of the nozzle undergoes deformation, which constrains the airflow and regulates its flow rate. To validate the feasibility of this design, we developed a 2D grid structure consisting of 4 × 13 square unit cells. This lattice integrates 329 adjustable members, and the members’ properties are consistent with those described in Section 2.2. By setting a specific target value for the continuous 13 reference points, which are located at the middle of the bottom edge of the structure, we can finally make the side produce a controllable curved deformation. Through the method, we have obtained the configuration that meets the target and verified its practicality through experimental testing and finite element simulations, as shown in Figure 6c. Compared with the overall decline of the initial configuration of Figure 6b after being driven, the channel of Figure 6c can be compressed and released from narrow to wide. The structure we designed here is an axisymmetric structure. As a rocket ascends, the external pressure on the engine nozzle increases. By allowing the nozzle's internal structure to deform in a controlled manner, as outlined in our design, the nozzle can adapt to varying atmospheric pressures. This adaptability enhances the success rate of rocket launches by optimizing nozzle performance under different altitude conditions, ultimately improving the overall efficiency and reliability of the propulsion system.

### 2.5 Inverse Design of Deformable Microstructures for Bio‐inspired Filtration Channel

Beyond aerospace, the inverse design holds promise for micro/nano‐scale biomedical devices.^[^ ^20^ ^]^ We propose a novel classification filtration channel, fabricated using micro/nano‐fabrication techniques (two‐photon polymerization, high‐resolution stereolithography) and advanced biomaterials (tunable hydrogels, biocompatible polymers like PCL or PLA),^[^ ^21^ ^]^ for applications such as cell separation/purification or blood filtration. This channel selectively captures and potentially stores target biological entities based on their physical properties by dynamically altering its internal geometry in response to fluid flow. The channel's architecture, determined by inverse design, dictates its filtration efficiency, selectivity, and capacity.

**Figure 7:**
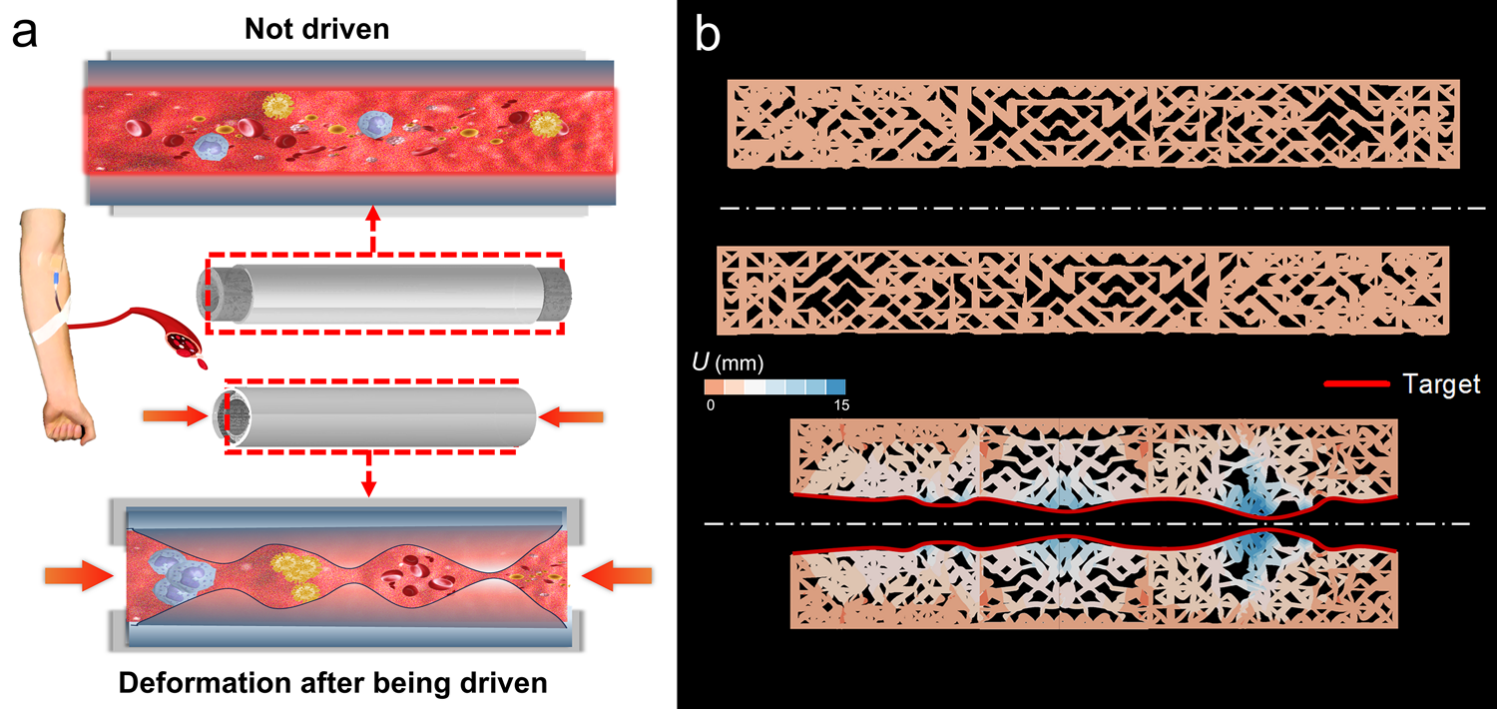
Inverse Design of Deformable Microstructures for Bio‐inspired Filtration Channel a) The potential application of a soft actuator in biomedical devices: the actuator is shown in two states—when not driven (top) and after being driven (bottom)—demonstrating its ability to deform and facilitate fluid flow in a biological environment. b) Simulation (depicted by the color gradient indicating displacement) results of a lattice metamaterial designed for targeted deformation, with the red line indicating the desired target shape.

As illustrated in **Figure** **7**a, the channel is designed to operate in two distinct states. In the absence of external loading, its internal filtration structure remains straight with even pores, facilitating free fluid passage. However, when external pressure is applied, the outer shell and internal structure deform. This deformation modulates pore size, enabling graded filtration based on particle size. To validate this design, we developed a microfluidic channel model as shown in Figure 7b. Utilizing inverse design, we established displacement targets for segmented deformation profiles of the channel wall under load. These targets were applied to critical nodes within functional regions. The channel is conceptualized as multiple independently optimized functional modules, each responsible for a specific filtration level. This modular approach is not only beneficial for design conceptualization but also serves as a key strategy for managing complexity and enhancing scalability. By breaking down a potentially large or intricate structure into smaller, computationally tractable units, the inverse design process can be applied more efficiently. These modules are then seamlessly integrated during fabrication to form the complete filtration channel, enabling controlled, non‐uniform deformation under driven. Finite element simulations confirm that the resulting configurations meet specific graded filtration requirements. The fluid‐actuated channel exhibits a highly complex and segmentally responsive structure, precisely forming desired narrowed and widened zones along its length, in contrast to the initial regular geometry.

By precisely programming the mechanical response of micro‐structured elements via inverse design, the channel autonomously forms desired pore sizes and distributions under specific flow conditions. This enables efficient, passive, and classified filtration and separation of target entities within the microfluidic device, potentially eliminating complex external actuation and opening avenues for advanced biomedical diagnostics and sample preparation. Furthermore, to address more complex structures and modeling challenges, integrating this method with Deep Learning (DL) techniques offers significant synergistic advantages. DL algorithms can predict optimal module configurations for large‐scale systems by learning from smaller‐scale simulations, bypassing resource‐intensive iterative optimizations. Our approach can generate robust datasets to enhance DL models’ ability to tackle intricate problems, thereby accelerating model development, reducing computational costs, and facilitating the design of sophisticated systems. This combination paves the way for broader design possibilities, potentially enabling hierarchical learning approaches and future automated design frameworks. Such synergy promises to significantly expand our capability to design complex, efficient, and generalizable systems, potentially revolutionizing fields like patient‐specific biomedical implant design.

## 3 Conclusion

In this research, we introduce a novel optimization approach for the inverse design of flexible, highly deformable mechanical metamaterials, significantly advancing traditional strategies by evaluating and controlling deformation behavior across material subregions. The approach, termed partitional semi‐random optimization, combines a statistical decision‐making procedure with a two‐step optimization scheme that integrates simulated annealing and genetic algorithm concepts. This method enhances local stiffness regulation, accelerates convergence by reducing redundant iterations, and expands the design space for homogeneous flexible materials, enabling the generation of structures capable of large, reversible deformations with customizable, multi‐objective deformation contours and paths. Demonstrated through successful simulation and experimental validation using 3D‐printed samples, including morphing wings and engine nozzles, this approach holds promise beyond aerospace and soft robotics, extending to micro/nano‐scale biomedical devices. For instance, it could enable flow‐actuated classification filtration channels fabricated via micro/nano‐techniques with advanced biomaterials, designed for applications like cell separation, where precisely controlled geometry changes facilitate selective capture. While the current study focuses on 2D designs, extending this robust and efficient framework to 3D offers significant potential for creating complex, multi‐dimensional shape‐morphing structures. Moreover, integrating this approach with deep learning techniques presents synergistic opportunities to address more complex modeling challenges, potentially enabling automated design frameworks for sophisticated systems such as patient‐specific implants, thereby revolutionizing fields requiring high adaptability in mechanical design.

## 4 Experimental Section

### Optimization Settings and Model Evaluation

In order to obtain the deformation of structures quickly and accurately, a customized code was used that imported the structure into ABAQUS in the form of a beam element to simulate the deformation and further feedback on the calculation results. The programs in 2.2 and 2.4 set up a grid‐based model to define and manipulate lines in a matrix. The grid consists of *n* (*n* is 13 in 2.2 and 2.4) vertical rows and *m* (*m* is 8 in 2.2 and 4 in 2.4) horizontal columns, with each line segment having a length of *l*. The model creates eight types of line segments, including horizontal and vertical lines, as well as diagonal lines in various orientations. The program initializes *n*+1 by *m*+1 cell arrays for storing line handles for each line type and assigns them specific lengths. It then iterates over the grid to generate these line segments, creating a complete grid layout. A complete grid layout will serve as an overall structure, and these lines will function as members that were adjusted and evolved during subsequent optimization to make topological and geometric adjustments to the structure. Finally, the validity of each line handle was verified, and the corresponding line code was written to a designated file. This file was then processed into ABAQUS modeling statements, which were subsequently imported into the ABAQUS script for simulation calculations. Within the ABAQUS script, a relevant set of reference points was established to ensure an accurate representation of the model for analysis. The displacement results obtained from the simulation were subsequently exported as a dataset and extracted specific values from the second column. These values were assigned to variables labeled v_1_ through v*_i_*, where *i* corresponds to the number of reference points considered. To evaluate the accuracy of these extracted values against predefined constants, a target metric *e* was calculated by summing the squared differences between the predefined constants and the extracted values. This metric serves as an objective measure to assess how closely the extracted values align with the predefined constants, providing a quantitative assessment of the simulation results’ accuracy.

The simulated annealing algorithm starts with an initial temperature of 100 and decreases it to a final temperature of 0.1, with a cooling factor of 0.95 determining the rate of temperature reduction. After the topological configuration of the member structure based on the partition probability selection was adjusted, the adjusted structure was evaluated and obtained an objective function value *e*. The process continues as long as the objective function value *e* is greater than 0 or the temperature is above the final threshold. The metropolis criterion, using the probability function

(4) $$p=\exp\left(\frac{e-e_{new}}{temperature}\right)$$

decides whether to accept a new, potentially worse solution to explore a broader solution space. The algorithm terminates when the objective function *e* is no longer greater than zero or the temperature falls below the final temperature, ensuring convergence toward an optimal or near‐optimal solution. The genetic algorithm was configured with specific settings: the max generations were set to 80, indicating the algorithm will run for up to 80 generations, and the population size was set to 30, which determines the number of individuals in each generation. The optimization was performed over *n* variables which was the number of existing members to the structure obtained after the previous step optimization. The array initializes with a default section width value of 0.8 for each member individual. During optimization, the GA function optimizes the section width values with constraints set between 0.5 and 2.0. The fitness function evaluated how well these section width values meet the target criteria by comparing computed results to a target value.

### Finite Element Analysis

The finite element simulations were conducted using ABAQUS 2023 software. The simulation assumes an elastic modulus of 25 MPa and a Poisson's ratio of 0.3, focusing on flexible materials without accounting for plastic yield or elastic buckling effects in detail. To ensure both computational efficiency and accuracy, a beam element model with a rectangular cross‐section of 0.8 × 0.8 mm was employed. The element type used was B31H, and the mesh size was set to 2.0 mm. Due to the complexity of the structure, the selected contact was a general contact. For the rectangular model, symmetry boundary conditions were applied along the *y*‐axis. The bottom of the model was fixed, while a vertical displacement was applied at the top to simulate the compression process. In the morphing wing model, clamping was enforced at both ends on the left side, and a displacement in the x‐direction was imposed on three central cells. The nozzle model had fixed ends in the *x*‐direction at the bottom, with the top boundary conditions consistent with those of the rectangular model. For the verification of designed structures, a 3D solid model matching the experimental specimen's dimensions was used, with element type C3D10 M and a mesh size ranging from 1.5 to 2.5 mm. The boundary conditions were similar to those described above, with the addition of constraints on *z*‐direction displacement. A 64‐core workstation, with CPU model INTEL Xeon Platinum 8358, was selected for calculations. The optimal number of cores for jobs was 8 and the number of jobs running at the same time was 16.

### Experimental Section

The specimens were fabricated by using 3D printing and mold shaping techniques, and the material was polyurethane rubber (The elastic modulus was 25 MPa.). This manufacturing technique effectively preserves the structural details. The dimensions of the rectangular specimen, comprising 8 × 13 cells, were ≈90 mm × 150 mm × 10 mm, with minor variations among individual specimens. The morphing wing test specimen size was ≈165 mm × 70 mm × 15 mm, while the nozzle test specimen size was ≈160 mm × 55 mm × 10 mm. In consideration of the 2D model utilized in the design, the experimental setup constrains displacement in the *z*‐direction by placing the specimen within a transparent acrylic plate container that had a width slightly larger than the specimen. To replicate the boundary conditions was the finite element simulation, screws, and porous plates were employed to secure the morphing wing and nozzle specimens, as illustrated in Figure S3 (Supporting Information). The compression experiments were carried out using a uniaxial testing machine and the experimental process and the deformations of the specimen were recorded with a camera.

## Conflict of Interest

The authors declare no conflict of interest.

## Supporting information



Supporting Information

Supplemental Movie 1

Supplemental Movie 2

Supplemental Movie 3

## Data Availability

The data that support the findings of this study are available from the corresponding author upon reasonable request.
