# Supplementary material for: Inverse Design of Highly Deformable Mechanical Metamaterial Based on Partitional Semi‐Random Optimization
Source: Adv Sci (Weinh). 2025 May 20;12(28):2415935. doi: 10.1002/advs.202415935 (PMC12302590; doi:10.1002/advs.202415935)
Supplement: Supplementary file 1 — Supporting Information [file ADVS-12-2415935-s004.docx]

Supporting Information

**Inverse Design of Flexible Lattice with Large Deformation Based on Partition Semi-random Screening Optimization**

*Xueqing Cao^1^, Zeang Zhao^1,*^, Panding Wang^1^, Shengyu Duan^1^, Hongshuai Lei^1^*

**This PDF file includes:**

- Supporting text information.
- Figure S1. The reference point displacement setting of the initial structure.
- Figure S2. The deformation of initial structure.
- Figure S3. Schematic of experimental equipment.
- Figure S4. Schematic of the lateral displacement and wing tip deformations.
- Figure S5. Schematic of the diagnosing structural rationality approach.
- Movie M1. Inverse Design of Regular Rectangular Structure with Different Customized Deformation Patterns
- Movie M2. Inverse Design of Deformation Path for Morphing Wing
- Movie M3. Inverse Design of Surface Deformation Patterns for Engine Nozzle


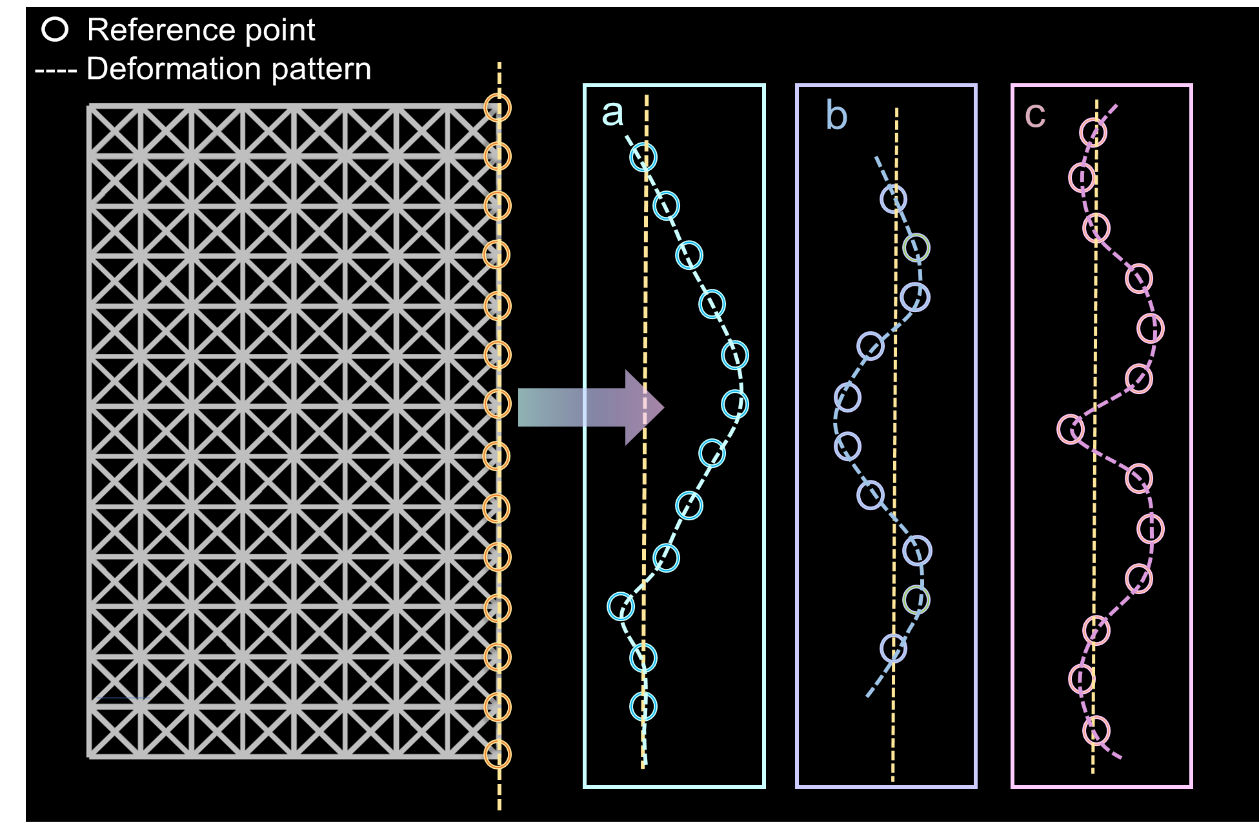


**Figure S1**. The reference point displacement setting of the initial structure. The grey diagram is the initial state of the structure, and the yellow circle represents the initial position of the reference point. the yellow dotted line is the initial deformation pattern of the structural reference point. a), b) and c) represent the target deformation position corresponding to the reference point under three different deformation patterns in 2.2.


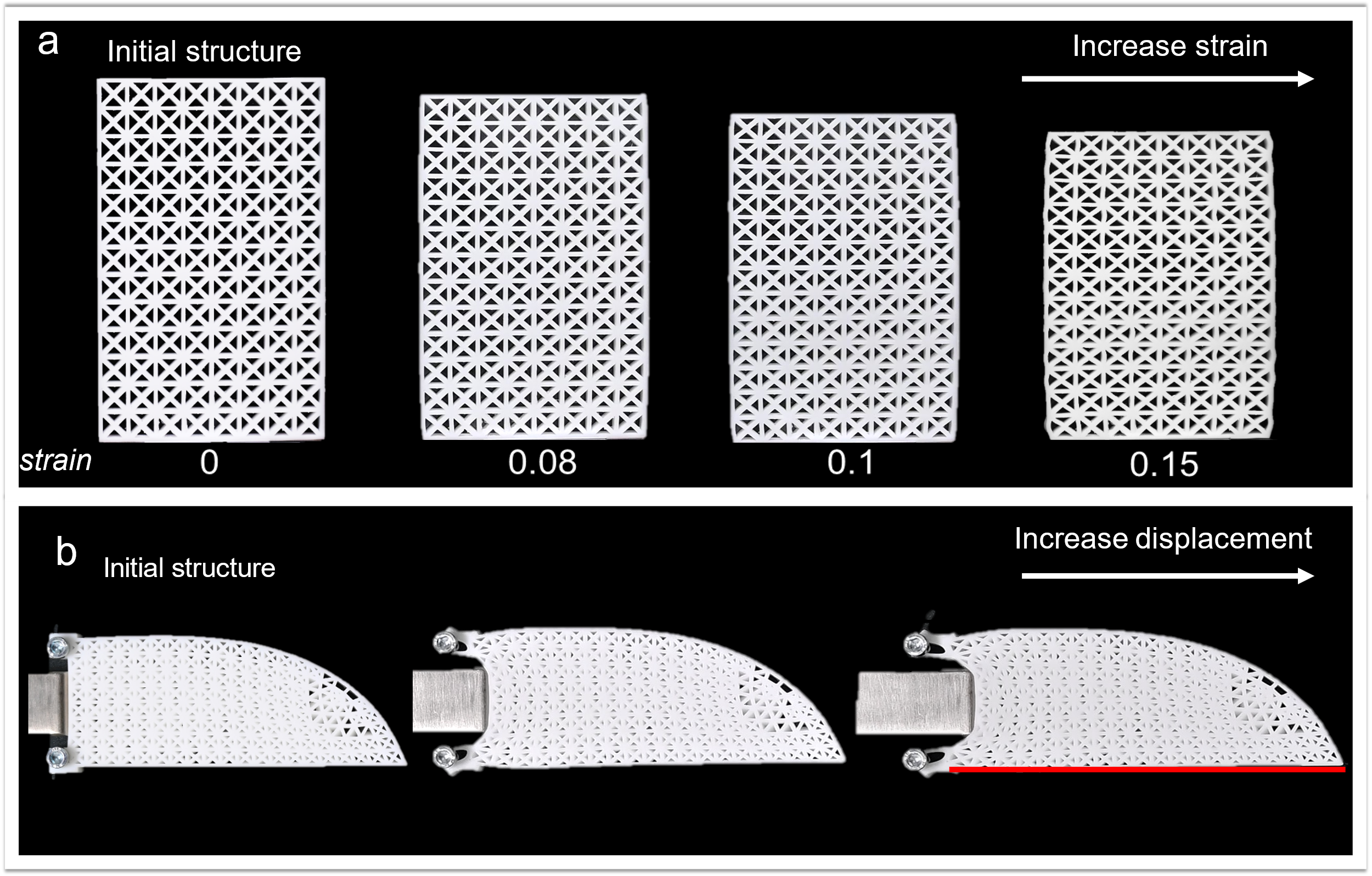


**Figure S2.** The deformation of initial structure. a) The deformation process of initial rectangular structure under compression. b) The deformation path of initial wing structure under driven.


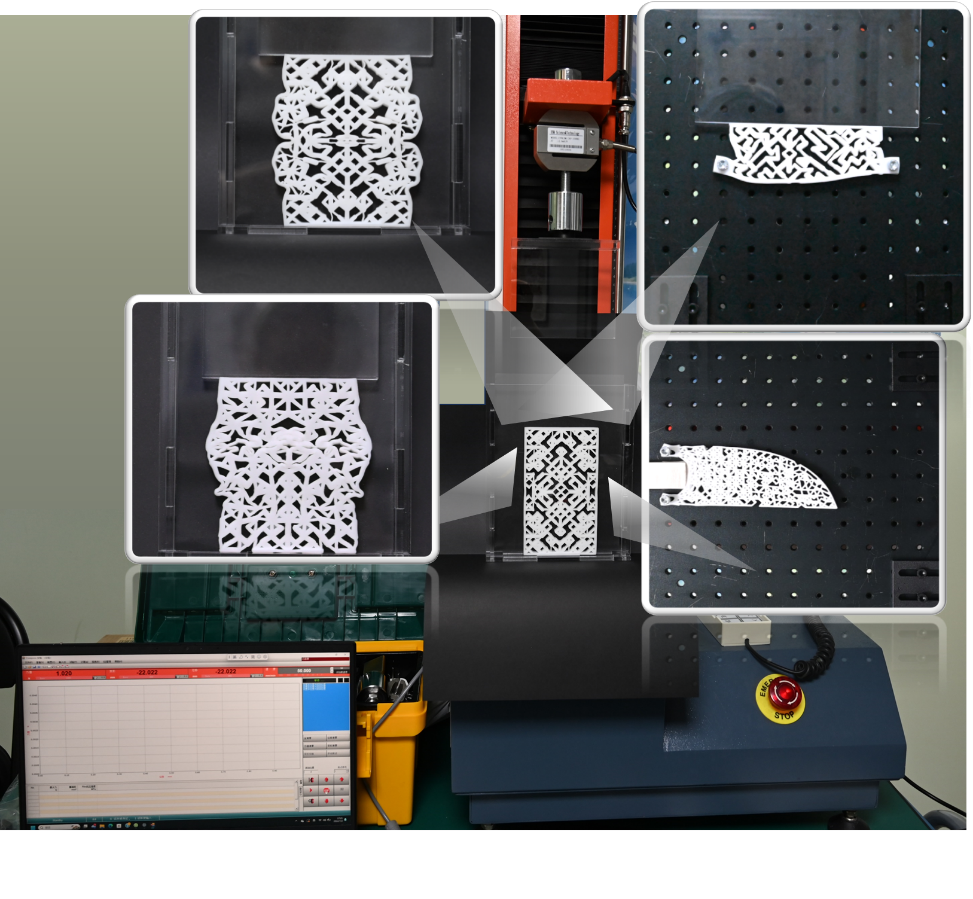


**Figure S3** Schematic of experimental equipment. The diagram shows the mechanical compression testing machine and the acrylic plate and porous plate for limiting displacement and the placement and loading methods of different types of models.


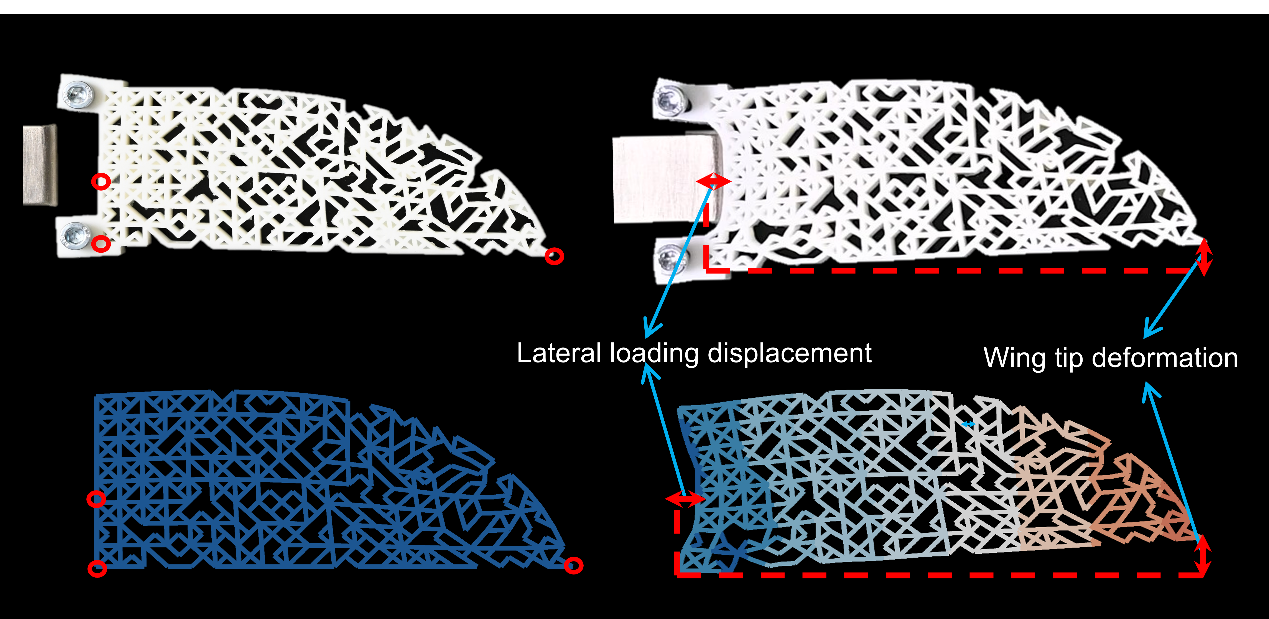


**Figure S4** Schematic of the position of the lateral displacement and wing tip deformations selection.

**S1 Materials and Methods**

**S1.1 The Progress of** **Diagnosing Structural Rationality**

When the structure is removed by a series of members, the structure tends to have some unreasonable (redundant) components, such as a small part of the structure isolated from the overall structure or these components may include isolated sections of the structure that are only connected at a single endpoint to other members, which consequently hinders effective load transmission. To address this issue and prevent the emergence of similar anomalies in the structure, we have developed specific programs to assess its rationality (Figure S5). The program employs two inspection methods to evaluate the structure. The first method focuses on identifying isolated components. It considers the unique characteristics of these segments—specifically, their lack of connection to the overall structure. We systematically analyze all nodes to determine whether any point within the structure cannot be reached from all other remaining points through the connected members. If such a point exists, it indicates that the removal of a members has resulted in an unreasonable configuration. In this case, the removed member is documented and reinstated. The second method examines the structural integrity concerning weak connectivity. This scenario often involves components that are linked to the overall structure via only one endpoint. We assess whether the coordinate values of all member endpoints within the structure are unique. If a member's endpoint coordinate appears only once, it signifies that the endpoint is not connected to any other members, indicating that the deletion of this member was inappropriate.


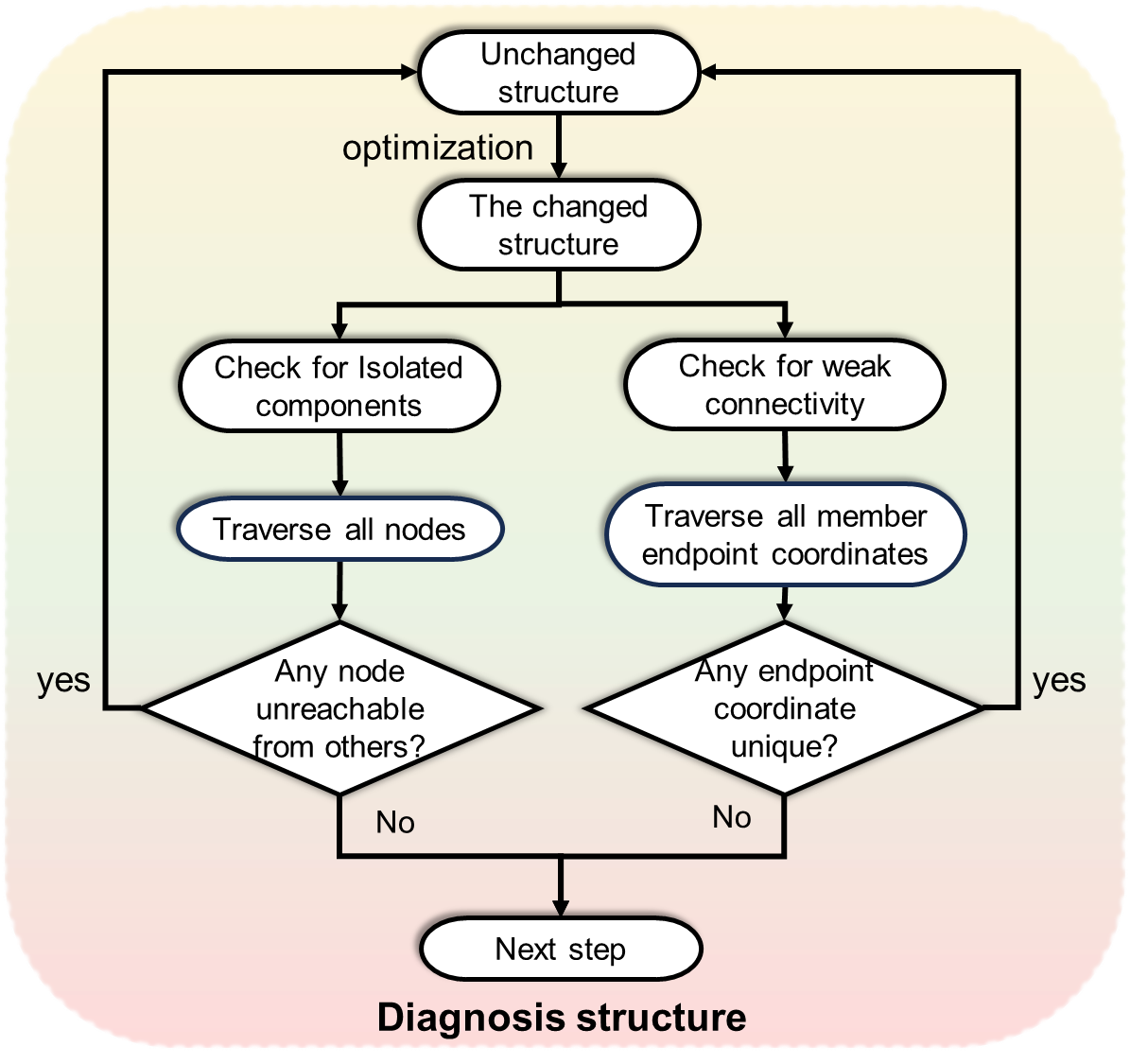


**Figure S5.** Schematic of the diagnosing structural rationality approach. The approach comprises ‘Check for Isolated Components’ and ‘Check for weak connectivity’. The first identifies isolated components by checking if any node is unreachable from others through connected members. The second checks for weak connectivity, assessing whether any endpoint is connected by only one member. If isolated or weakly connected components are found, it will be restored to the structure before the change.

**S1.2 Simulated Annealing Algorithm**

The simulated annealing algorithm is a stochastic optimization method inspired by the physical annealing process, aiming to find a global optimum. It mimics the heating and slow cooling of metals. The algorithm starts with a random solution and generates new solutions through small random perturbations. If the new solution has a better objective function value, it is accepted; if worse, it may still be accepted based on a temperature parameter and an acceptance probability (typically using the Metropolis criterion). As iterations progress, the temperature decreases, reducing the likelihood of accepting worse solutions and guiding the process toward a stable optimum. Simulated annealing is effective for combinatorial optimization problems, such as the traveling salesman problem and scheduling tasks, due to its strong global search capability.

The Simulated Annealing in our paper is used to optimize a specific objective function ***e***, which is calculated based on data from $e=\sum_{k=1}^{N} \left(U_{k}-U_{k}^{*}\right)^{2},k=1,\ldots,K$. where $U_{k}$ and $U_{k}^{*}$ are the simulation and target induced displacements at selected reference points, respectively, *K* is the total number of reference points. According to the results of the partition evaluation, we will set different probabilities for each subregion to be selected. At the beginning of the iteration, the higher the probability, the easier the members in the subregion is selected. The algorithm iterates by making random modifications to the data and evaluates the new objective value $e\_new$. We aim to maximize it by minimizing the cost function Δ = −*e*. This minimization process utilizes the Monte Carlo method alongside an optimization algorithm. In each iteration, starting from the current configuration with Δ = Δ0, a trial configuration is generated by randomly removing or re-adding a beam. Input and output nodes are excluded from pruning, as well as any nodes that are constrained from movement (frozen). If $e\_new$ is better (lower), the modification is accepted. If worse, it may still be accepted with a probability that decreases as the temperature reduces. This probabilistic acceptance helps explore a broader solution space before gradually focusing on better solutions.

**S1.3 Genetic Algorithms for Topology Optimization**

Genetic Algorithm is employed to optimize an array of cross-section values to minimize the error in a specific target function. Genetic Algorithm is employed to optimize an array of cross-section values to minimize the error in a specific target function. The structure optimized by genetic algorithm is the final topological configuration obtained by the previous annealing algorithm. Before the optimization, the members of the structure will be formatted again. The code starts by initializing an array of cross-section values with a default of 0.8. Then the objective function value *e* under the initial setting is calculated to provide a reference for subsequent genetic variation. It then sets up the Genetic Algorithm parameters, including the maximum number of generations, population size, and display options.

The core of the Genetic Algorithm is embedded in a loop that runs for 80 generations. During each generation, the ga function from MATLAB's Optimization Toolbox is used to perform the optimization. This function is configured with specific options such as the number of generations and the size of the population. The fitness_function evaluates the performance of each individual in the population by comparing the calculated error against a target value.

Each generation's results are logged in a file for tracking the progress of the optimization, capturing details such as the best fitness value and average fitness value. The overall goal is to iteratively improve the radii values so that the model's output closely matches the target value, ultimately achieving the best possible configuration through the evolutionary process.
